# Distribution and Risk Factors of Hepatitis B Virus Infection in the General Population of Central Iran

**DOI:** 10.5812/hepatmon.822

**Published:** 2012-02-29

**Authors:** Mohammad Reza Ghadir, Mojtaba Belbasi, Akram Heidari, Mahboobeh Jandagh, Iman Ahmadi, Hosseinali Habibinejad, Alireza Kabiri, Amir Hossein Ghanooni, Abolfazl Iranikhah, Seyed Moayed Alavian

**Affiliations:** 1 Department of Gastroenterology, Faculty of Medicine, Qom University of Medical Sciences, Qom, IR Iran; 2 Iranian Blood Transfusion Organization Research Center, Qom, IR Iran; 3 Faculty of Medicine, Qom University of Medical Sciences, Qom, IR Iran; 4 Qom University of Medical Sciences, Qom, IR Iran; 5 Iran National Science Foundation, Tehran, IR Iran; 6 Department of Pediatrics, Faculty of Medicine, Qom University of Medical Sciences, Qom, IR Iran; 7 Baqiyatallah Research Center for Gastroentrology and Liver Diseases, Tehran, IR Iran

**Keywords:** Hepatitis B, Prevalence, Iran

## Abstract

**Background:**

Hepatitis B is the most common chronic viral infection in humans and the most common cause of death among viral hepatitis. As 70% to 80% of chronic hepatitis cases are caused by HBV in Iran, this virus alone is considered the most important cause of liver diseases and the major cause of mortality arising from viral hepatitis cases in Iran.

**Objectives:**

We planned this study to determine the prevalence of hepatitis B in the general population of Qom, central Iran.

**Patients and Methods:**

The present study is a cross-sectional study. A total of 3690 samples were collected from 7 rural clusters and 116 urban clusters. Ten teams, each consisting of 2 trained members, were assigned to conduct the sampling and fill the questionnaires. The data were analyzed using SPSS.

**Results:**

The prevalence rate of hepatitis B infection in Qom Province was 1.3%. The mean age of the patients with hepatitis B was 44.17 years. The prevalence of hepatitis B was 1.6% in men and 1.1% in women. Moreover, the prevalence of hepatitis B correlated positively with age, tattooing, and literacy level.

**Conclusions:**

The prevalence rate of hepatitis B in Qom is 1.3%. It is possible to prevent the disease by increasing public awareness. Further investigation on clinical presentations and a determination of the genotype of the virus are suggested.

## 1. Background

Hepatitis B is the most common chronic viral infection in humans. HBV infection is one of the major public health problems throughout the world. Nearly 350 to 400 million people suffer from this infection globally, and 1 million people per year lose their lives due to complications of this infection [1]. On the other hand, hepatitis is the major cause of hepatic cirrhosis and hepatocellular carcinoma. It is believed that the most common cause of liver cancers is viral infection. The prevalence of hepatitis B carriers in different parts of the world varies from 0.5% to > 8%. Since most children and adults suffering from this virus do not have clinical symptoms, seroepidemiological studies provide a broader picture of the distribution of the infection. Determining the trend of HBV infection is important in assessing the efficiency of vaccination in infants and young adults [2][3][4]. In the studies that have been conducted in Iran, the average rate of HBV infection has always been 3%. In these studies, the rate of HBV prevalence in blood donors is 3.4%, ranging from 1.7% in Fars Province to over 5% in Sistan-Baluchestan Province [2][3]. This difference has also been seen in recent studies, in which the HBV prevalence rate in 2003 was 0.5% in blood donors and students [5][6] and 6% in large vehicle drivers [7]. In a study conducted by the Gastroenterological and Liver Diseases Research Center of Tehran University in Gonbad, northeastern Iran, the prevalence of HBV in normal inhabitants of the city was reported to be 4.5%-1.5 times the average rate [8]. The high prevalence rate shows that despite the 35-year history of studies on this disease in Iran and despite the nationwide immunization of newborns, infection with this virus is still considered a major health problem in Iran, and the risk of severe complications poses a threat to many individuals in the community.

## 2. Objectives

Since the studies in Iran have been based on serological tests on individuals visiting health centers and blood donors-not on normal individuals in the community-because the policies of the Blood Transfusion Organization (BTO) for selecting blood donors have been changed in recent years, and because the figures of 3% [3] and 5% [5] obtained in recent studies conducted by the BTO cannot show the actual prevalence rate of the infection in the community, the present study aimed to determine the prevalence rate and risk factors of HBV infection in Qom, central Iran.

## 3. Patients and Methods

The present study is a cross-sectional study. A population of 3690 individuals was assigned to the study by cluster sampling. Seven rural clusters and 116 urban clusters were selected, based on girls' elementary schools in different districts of the province. For sampling purposes, only one person from each family was selected randomly by draw of the lots. Then, the phases of the study were explained to the individuals who were eligible for the study. Those who were interested were invited to undergo tests and complete the questionnaires. A questionnaire, including such data as age, sex, marital status, occupation, literacy level, blood transfusion history, history of surgery, dental surgery, viral hepatitis, immunization, narcotic drug consumption, smoking, tattooing, cupping, endoscopy, and AIDS, was completed for each participant. Ten teams consisted of two trained members who were responsible for preparing samples and completing the questionnaires. Nearly 5 cc of serum was taken from each subject and transferred to the lab under sterile conditions in ice bags. HBs antigen was measured by ELISA. All patients gave written informed consent, and the study protocol was approved by the ethics committee of Qom University of Medical Sciences. Quantitative variables were analyzed by t test and Mann-Whitney test. Categorical variables were analyzed by Pearson chisquare test and Fisher exact test. Multivariate analysis was performed to adjust for potential confounders. Statistical analysis was performed using SPSS®. P < 0.05 was considered significant.

## 4. Results

Out of 3690 subjects, 48 (1.3%) suffered from hepatitis B, and 46.6% of subjects were male and 54.4% was female (Table 1); 129 (3.5%) subjects had a history of immunization, and none had hepatitis B. Further, 1.3% of subjects without an immunization history suffered from hepatitis B. The occupational distribution of the participants is presented in Table 2. In this study, the mean age of subjects with hepatitis B was 44.17 years, with a standard deviation of 15.75 years; the mean age of those who did not suffer from hepatitis B was 36.45 years, with a standard deviation of 14.42 years, which, by Kolmogorov test, showed a significant difference from normal distribution. Mann-Whitney test was used to investigate the relationship between age and hepatitis B infection. As a result, the age of the subjects with and without hepatitis B infection differed significantly (P = 0.001). Therefore, we concluded that the mean age of individuals with hepatitis was significantly higher than those who did not suffer from the disease. Of all samples, 0.7% (26 subjects) had a history of viral hepatitis, 7.7% of whom had suffered from hepatitis B. Of 48 subjects with hepatitis B, 47 answered this question, 4.3% of whom had a history of viral hepatitis, and of 3444 subjects without hepatitis, 7% had a history of hepatitis B. In this study, the prevalence of smoking in Qom Province was 9% (those who had a history of smoking at least 1 month prior to the study were considered smokers). The prevalence of hepatitis B infection was 2.2% in smokers versus 1.2% in nonsmokers. Two percent of the statistical population in this study had rarely used alcohol, and 4.3% of individuals with hepatitis B in Qom Province had rarely used alcohol. The prevalence of hepatitis B in such individuals was 2.7%, versus 1.3% in those who had never used alcohol. By chi-square test (P = 0.008), there was a significant relationship between tattooing and hepatitis B infection. The prevalence of HBV infection by occupation of the participants is presented in Table 2. By chi-square test, there was a significant relationship between the level of education and hepatitis B (P = 0.019). Among the subjects, 0.4% had AIDS, none of whom had hepatitis B infection. The Fisher test showed no significant difference (P = 1.000). Multivariate logistic regression was performed to adjust for potential confounders, and the results are presented in Table 3.

**Table 1:** Seoprevalence of HBs Antigen According to Demographic Characteristics in Qom

	**Total Population, No. (%)**	**HBsAg Positive, No. (%)**	**HBsAg Negative, No. (%)**
Sex			
Male	1709 (46.62)	27 (1.58)	1682 (98.42)
Female	1957 (53.38)	21 (1.07)	1936 (98.93)
History of hepatitis			
Yes	47 (1.35)	23 (48.94)	24 (51.06)
No	3444 (98.65)	45 (1.31)	3420 (99.30)
Drug addiction			
Yes	67 (1.87)	0 (0.00)	67 (100.00)
Occasionally	20 (0.56)	2 (10.00)	18 (90.00)
No	3498 (97.57)	44 (1.26)	3454 (98.74)
Smoking			
Yes	325 (9.01)	7 (2.15)	318 (97.85)
No	3282 (90.99)	41 (1.25)	3241 (98.75)
Tattooing			
Yes	166 (4.54)	6 (3.61)	160 (96.39)
No	3490 (95.46)	42 (1.20)	3448 (98.80)
Marital status			
Single	677 (19.09)	6 (0.89)	671 (99.11)
Married	2741 (77.28)	38 (1.39)	2703 (98.61)
Divorced	16 (0.45)	1 (6.25)	15 (93.75)
Widowed	113 (3.19)	2 (1.77)	111 (98.23)
Education			
None	474 (12.96)	14 (2.95)	460 (97.05)
Writing and reading	231 (6.32)	3 (1.30)	228 (98.70)
Primary	690 (18.87)	11 (1.59)	679 (98.41)
Middle	661 (18.08)	7 (1.06)	654 (98.94)
High school	942 (25.77)	6 (0.64)	936 (99.36)
Academic	507 (13.87)	5 (0.99)	502 (99.01)
Seminary	151 (4.13)	1 (0.66)	150 (99.34)
Transfusion history			
Yes	123 (3.40)	2 (1.63)	121 (98.37)
No	3498 (96.60)	45 (1.29)	3453 (98.71)
Dialysis history			
Yes	11 (0.31)	0 (0.00)	11 (100.00)
No	3568 (99.69)	47 (1.32)	3521 (98.68)
Surgery history			
Yes	114 (3.21)	1 (0.88)	113 (99.12)
No	3441 (96.79)	45 (1.31)	3396 (98.69)
Cupping history			
Yes	795 (21.73)	6 (0.75)	789 (99.25)
No	2863 (78.27)	42 (1.47)	2821 (98.53)
Dental procedure history			
Yes	2238 (60.85)	29 (1.30)	2209 (98.70)
No	1440 (39.15)	19 (1.32)	1421 (98.68)
Endoscopic history			
Yes	281 (7.64)	7 (2.49)	274 (97.51)
No	3396 (92.36)	41 (1.21)	3355 (98.79)

**Table 2:** Prevalence of HBV Infection According to Occupation of the Participants in Qom

	**Total Subjects, %**	**HBV Infection Prevalence, %**
School-goers	5.3	0
University students	5.1	0.5
Homemakers	46.3	1
Farmers	1.3	2.1
Employees	8.1	1
Clerics	4.2	0.6
Self-employed	17.8	2
Prisoners	3.5	3
Soldiers	0.2	0
Unemployed	2.6	2.1
Workers	5.2	0
Other jobs	0.2	0

**Table 3:** Multivariate Logistic Regression of Possible Risk Factors For Hbs Antigen

	**HBsAg Positive**	
	**P value**	**Statistically Significant**
Sex	0.178	No
Age	0.001	Yes
History of hepatitis	0.047	No
Smoking	0.175	No
Tattooing	0.008	Yes
Marital status	0.023	Yes
Education	0.019	Yes
Transfusion history	0.744	No
Dialysis history	1	No
Surgery history	0.6	No
Cupping history	0.118	No
Dental procedure history	0.951	No
Endoscopic history	0.68	No

## 5. Discussion

Despite the availability of an effective vaccine and the progress that has been made in antiviral treatments, hepatitis B continues to be a major public health problem in the world [9]. The present study found that the prevalence of hepatitis B in the Qom Province is 1.3%. The studies conducted by Ataei et al. in Esfahan, Iran, and Kazerani in Kermanshah, Iran, also observed a rate of 1.3%, which confirms the results of our study [10][11]. In a study by Alavian et al. the prevalence of hepatitis B in Iran was 2.14%. In this study, the geographical distribution of hepatitis B in Iran was heterogeneous, indicative of the difference between likely risk factors in different parts of Iran [12]. The prevalence of hepatitis B is 0.5% to 0.8% in Australia [13] and 10% to 19.5% in Vietnam [14][15]. The difference in the prevalence of hepatitis B between different countries is probably indicative of the disparate rates of contact with risk factors. In this study, the prevalence of hepatitis B in males and females was 1.6% and 1.1%, respectively. In the studies by Alizadeh et al. in Hamedan Province, Iran, and Meraat et al. in Hormozgan Province, Iran, the prevalence in males was higher than in females, which confirms the results of our study [16]. The higher rate of hepatitis B prevalence in males than females is likely due to the higher exposure to occupational HBV risk factors in men in Iran. In several studies in Iran, females had a higher prevalence [17], and in others, the prevalence in males and females was equal [18][19].

In our study, the mean age of patients with hepatitis B infection was 44.17 years. In the study by Qureshi et al. in Pakistan, the highest rate of hepatitis B correlated with age above 50 years [20]. In the study by Zali et al. the highest prevalence also occurred in the 50-59-year age group [21]. However, in the study by Abdollahi et al., the highest prevalence in Golestan Province was observed in the 25-34-year age group [22]. In the present study, 1.9% of the subjects had a history of taking narcotics, of which 21.4% were intravenous users. The prevalence of HBV in individuals who took narcotics occasionally was higher than those who had no history. In the study by Qureshi et al., the highest prevalence was in the group who had a history of over 10 injections, and as the number of injections increased, so did the rate of HBV [20]. In this study, the prevalence of HBV was higher in divorced individuals. There was a significant relationship between HBV prevalence and marital status (P = 0.023). In the study by Abdollahi et al. the rate of HBV was higher in singles than those who had married at least once [22]. In the study by Zali et al. marital status was also a key indicator of prevalence [21]. In many studies, marriage and heterosexual relationships are considered risk factors for HBV [23][24][25]. Such differences are probably due to cultural differences in each community. In a study by McQuillan et al. in the US, the HBV was higher in widows than others [26].

In this study, the prevalence of HBV in illiterates was higher than in others, and there was a significant relationship between literacy level (education) and hepatitis B. In a study by McQuillan et al. the highest rate of HBV was in literates with below-high-school qualifications, and HBV prevalence fell with rises in literacy level, which corresponds to the results of our study [26]. In the present study, the prevalence of HBV was lower in individuals with a history of surgery than those who did not have a history, whereas in the study by Qureshi et al. in Pakistan, the rate of HBV in individuals with a history of surgery was higher [20]. This is probably indicative of better postoperative care in Iran. In a study by Zali et al. a history of major surgery was a risk factor for HBV [21].

The prevalence of HBV in individuals with a history of tattooing was significantly higher than those without a history. In a study by Zhang et al. in Canada, tattooing did not increase the risk of hepatitis significantly [27]. In a study by Qureshi et al. in Pakistan, the prevalence of HBV in individuals with a history of tattooing was not significantly higher than those without a history of tattooing [20]. This difference might be due to the effect of the confounding variable in our study. In most studies in Iran, a history of blood transfusion has not been a major risk factor, which corresponds to our study, whereas other factors, such as sexual relationship, injections, imprisonment, driving, and non-sterile actions, such as tattooing, play a more important role in communicating hepatitis B [28]. The study by Zali et al. considered such risk factors as old age, masculinity, marital status, history of contact with hepatitis, sexual activity, intravenous drug use, major surgeries, visiting unqualified dentists, and certain occupations [29]. Despite the fact that there is no effective treatment available that can, in the case of early diagnosis, inhibit the trend of the acute disease, some treatment procedures can stop the liver deterioration and prevent the incidence of hepatic cirrhosis and hepatocellular carcinoma. Therefore, faster identification of chronic cases without clinical symptoms, which can be done by taking a history, conducting serological tests, measuring hepatic enzymes, measuring viral load, performing ultrasound sonography, and determining the risk factors that lead to the disease in the region, can help officials and authorities adopt proper measures to identify the virus-transmitting factors in the community with the aim of cutting the transmission chain.

The fate of chronic hepatitis B disease from the carrier who has no symptoms to the emergence of compensated or decompensated cirrhosis and/or hepatocellular carcinoma and, ultimately, death can vary. The emergence of the complications of the disease can lead to long periods of hospitalization, consumption of expensive medications, and ultimately liver transplantation, which imposes heavy costs on the government and the community. Thus, the virus-transmitting factors in the community and the chronic carriers of the virus must be identified all countries, which has been considered in other developing countries. The limitation of our study was that we did not measure the serum levels of HBc antigen in participants of this study; thus, our results may be underestimated. Based on this study, the prevalence rate of hepatitis B in Qom Province, Iran, was 1.3%. Further investigations on the clinical presentations and genotype of the virus are recommended. Since this study was conducted for the first time in the region, the results should provide officials with new and comprehensive data on the characteristics of the disease in the region, allowing them to make proper plans and adopt appropriate strategies in preventing, treating, and immunizing patients.
